# Complementary Role of CEUS and CT/MR LI-RADS for Diagnosis of Recurrent HCC

**DOI:** 10.3390/cancers15245743

**Published:** 2023-12-07

**Authors:** Mei-Qing Cheng, Hui Huang, Si-Min Ruan, Ping Xu, Wen-Juan Tong, Dan-Ni He, Yang Huang, Man-Xia Lin, Ming-De Lu, Ming Kuang, Wei Wang, Shao-Hong Wu, Li-Da Chen

**Affiliations:** 1 Department of Medical Ultrasonics, Institute of Diagnostic and Interventional Ultrasound, The First Affiliated Hospital of Sun Yat-sen University, Guangzhou 510080, China; chengmq@mail.sysu.edu.cn (M.-Q.C.); huangh233@mail.sysu.edu.cn (H.H.); ruansm@mail2.sysu.edu.cn (S.-M.R.); tongwj@mail.sysu.edu.cn (W.-J.T.); huangy268@mail.sysu.edu.cn (Y.H.); linmxia@mail.sysu.edu.cn (M.-X.L.); lumd@live.com (M.-D.L.); kuangm@mail.sysu.edu.cn (M.K.); wangw73@mail.sysu.edu.cn (W.W.); 2 Department of Radiology, The First Affiliated Hospital of Sun Yat-sen University, Guangzhou 510080, China; xuping3@mail.sysu.edu.cn; 3 Department of Medical Ultrasonics, The Seventh Affiliated Hospital of Sun Yat-sen University, Shenzhen 518107, China; hedn5@mail2.sysu.edu.cn; 4 Department of Hepatobiliary Surgery, The First Affiliated Hospital of Sun Yat-sen University, Guangzhou 510080, China

**Keywords:** hepatocellular carcinoma, ultrasonography, computed tomography, magnetic resonance imaging, contrast agent

## Abstract

**Simple Summary:**

The diagnostic performance of LI-RADS on CEUS and CT/MRI for characterizing primary HCC has been widely evaluated, but their diagnostic performance on recurrent HCC has not yet. Previous studies that used the same diagnostic criteria for the diagnosis of recurrent HCC proved inadequate, as only a subset (approximately 55–80%) of recurrent HCC cases exhibited the typical imaging features observed in primary HCC on contrast-enhanced CT or MRI. Consequently, this study embarked on an investigation to establish the optimal diagnostic algorithms for recurrent HCC using CEUS, CT, and MRI, and compared the diagnostic performance of CEUS and CT/MRI LI-RADS for recurrent HCC. The results indicated that LR-4/5/M criteria were optimal for CEUS and CT, while LR-5/M criteria were suitable for MRI. CEUS demonstrated better diagnostic performance than CT, while was comparable to MRI. Further, this study analyzed factors affecting the accurate characterization and detection of recurrent HCC on CEUS. Depth and visualization score C were found to hinder accurate characterization, and lesions in US blind spots with visualization score C posed challenges for detection.

**Abstract:**

Purpose: We retrospectively compared the diagnostic performance of contrast-enhanced ultrasonography (CEUS) and contrast-enhanced computer tomography–magnetic resonance imaging (CT/MRI) for recurrent hepatocellular carcinoma (HCC) after curative treatment. Materials and methods: After curative treatment with 421 ultrasound (US) detected lesions, 303 HCC patients underwent both CEUS and CT/MRI. Each lesion was assigned a Liver Imaging Reporting and Data System (LI-RADS) category according to CEUS and CT/MRI LI-RADS. Receiver-operating characteristic (ROC) curves were computed to determine the optimal diagnosis algorithms for CEUS, CT and MRI. The diagnostic accuracy, sensitivity, specificity, and area under the curve (AUC) were compared between CEUS and CT/MRI. Results: Among the 421 lesions, 218 were diagnosed as recurrent HCC, whereas 203 lesions were diagnosed as benign. In recurrent HCC, CEUS detected more arterial hyperenhancement (APHE) and washout than CT and more APHE than MRI. CEUS yielded better diagnostic performance than CT (AUC: 0.981 vs. 0.958) (*p* = 0.024) comparable diagnostic performance to MRI (AUC: 0.952 vs. 0.933) (*p* > 0.05) when using their optimal diagnostic criteria. CEUS missed 12 recurrent HCCs, CT missed one, and MRI missed none. The detection rate of recurrent HCC on CEUS (94.8%, 218/230) was lower than that on CT/MRI (99.6%, 259/260) (*p* = 0.001). Lesions located on the US blind spots and visualization score C would hinder the ability of CEUS to detect recurrent HCC. Conclusion: CEUS demonstrated excellent diagnostic performance but an inferior detection rate for recurrent HCC. CEUS and CT/MRI played a complementary role in the detection and characterization of recurrent HCC.

## 1. Introduction

Hepatocellular carcinoma (HCC) is the sixth-most common malignant tumor and ranks fourth in cancer-related deaths worldwide [1]. Despite curative therapy such as surgical resection and thermal ablation, the 5-year post-treatment recurrence rate remains at 70% [2]. The high frequency of recurrence as well as the more aggressive characteristics [3] of recurrent HCC contribute to poor long-term overall survival and heavy social burden, which draw great concern for HCC management. Accurate detection and characterization of HCC recurrence allowing timely salvage therapy are of paramount significance.

Imaging modality is essential for the detection and characterization of recurrent HCC. The American College of Radiology (ACR) established the Liver Imaging Reporting and Data System (LI-RADS) of contrast-enhanced computer tomography–magnetic resonance imaging (CT/MRI) or contrast-enhanced ultrasonography (CEUS) for liver nodule categorization [4,5], which standardized the diagnostic criteria for both primary and recurrent HCC. For primary HCC, previous studies indicated that using LI-RADS category 5 (LR-5) as a diagnostic criterion yielded nearly 100% specificity for liver nodule characterization, which allowed non-invasive diagnosis [6,7]. In recurrent HCC diagnosis, accuracy may be more appreciated, as a considerable portion of newly developed nodules are likely to be HCC. However, few studies have evaluated the application of the LI-RADS system in diagnosing recurrent HCC. Wang et al. evaluated the diagnostic performance of MRI LI-RADS in characterizing recurrent HCC [8]. The diagnostic performance was not desirable, with accuracy of 55.8% when using LR-5 as diagnostic criteria, indicating that LR-5 diagnosis criteria or this imaging modality may not be optimal for recurrent HCC diagnosis. This low accuracy may be attributed to the atypical enhancement pattern of recurrent HCC [8,9,10], with only approximately 55–80% of recurrent HCC demonstrating typical imaging features on CT or MRI in previous studies [8,11,12,13]. Growing evidence suggests that CEUS has unique advantages in diagnosing recurrent HCC and the probability of detecting arterial hyperenhancement (APHE) and washout on equivocal CT/MRI nodules [12]. Its high-contrast resolution and temporal resolution together with the pure use of intravascular agents offer a continuous real-time detection of APHE and real washout [14], which seemed promising in the accurate diagnosis of recurrent HCC. No studies have ever evaluated the diagnostic accuracy of CEUS LI-RADS for diagnosing recurrent HCC.

This study was designed to investigate the optimal diagnosis criterion in recurrent HCC on CEUS LI-RADS and CT/MRI LI-RADS and subsequently compared the diagnostic performance of CEUS LI-RADS and CT/MRI LI-RADS, and also the detection rate of these three modalities to find the most appropriate diagnostic modality in clinical practice.

## 2. Materials and Methods

This retrospective cohort study was approved by the institutional review board and the requirement for written informed consent was waived for all patients.

### 2.1. Patients

We recruited all patients with a history of HCC successfully treated with curative therapy (surgery and/or thermal ablation) from July 2014 to December 2018 who met the following inclusion and exclusion criteria during follow-up. Patients underwent contrast-enhanced imaging and AFP at least every 3–6 months for the first 2 years after curative treatment and every 6–12 months thereafter, with consideration of shorter intervals during the first year given a higher risk of recurrence during that time.

**Figure 1:**
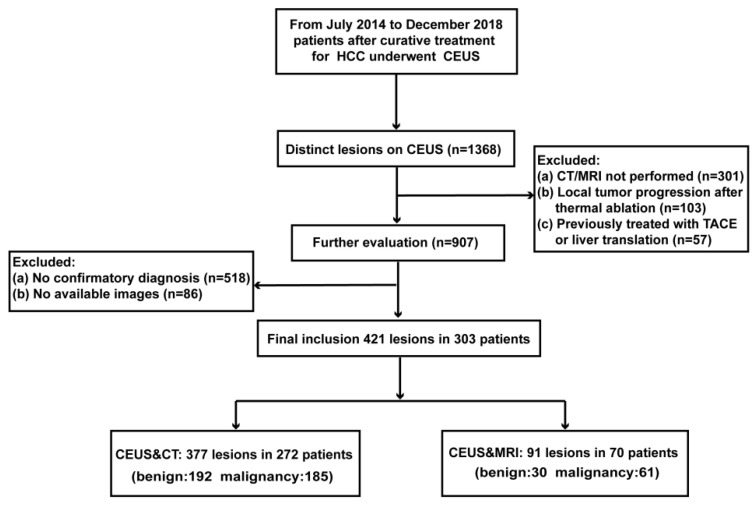
Flow diagram of participant enrollment. Note: A total of 303 patients with 421 lesions underwent CEUS, 272 patients with 377 lesions underwent both CT and CEUS, and 70 patients with 91 lesions underwent both MRI and CEUS. Among these patients, 39 patients with 47 lesions underwent CEUS, CT and MRI.

The eligibility criteria for participants’ enrollment were (a) patients with focal liver lesions detected on CEUS and (b) contrast-enhanced CT or MRI scans within 2 weeks of CEUS. The exclusion criteria were (a) local tumor progression after thermal ablation, (b) lesions previously treated with TACE or liver transplantation, (c) no eligible reference standard (described in the Reference standard section), and (d) no available images (CEUS or CT/MRI). A detailed flowchart describing patient selection is presented in Figure 1.

### 2.2. Image Archiving and Scanning Parameters of CEUS, CT and MRI

Equipment for CEUS: an Aplio 500 machine (Toshiba Medical Systems, Tokyo, Japan) with a 375 BT convex transducer (frequency range, 1.9–6.0 MHz) or an Aixplorer Ultrasound system (SuperSonic Imagine, Aix-en-Provence, France) equipped with an SC6-1 convex probe (frequency range, 1.0–6.0 MHz).

Equipment for CT: a 64-detector row (Aquilion CXL, Toshiba Medical System, Tokyo, Japan) or a 320-detector row CT machine (Aquilion One, Toshiba Medical System, Tokyo, Japan).

Equipment for MRI: a 3.0 T MR system (SIGNA Pioneer, GE Healthcare, Milwaukee, WI, USA) or 3.0 T MR system (Magnetom Verio, Siemens Healthineers, Erlangen, Germany).

Imaging archiving and scanning parameters of CEUS, CT and MRI examinations are described in Supplementary Material S1.

### 2.3. Imaging Analysis

Two radiologists (M.Q.C. and S.H.W.), each with more than 10 years of experience in hepatic imaging and who were blinded to pathological results and clinical or laboratory information, independently reviewed the CEUS, CT and MRI images. When disagreements arose, the third radiologist (L.D.C.), with 15 years of experience in liver imaging, participated in further evaluation until a consensus was reached. Each nodule was categorized based on the major and ancillary imaging features of the nodules according to CEUS LI-RADS version 2017 and CT/MRI LI-RADS version 2018 [4,15].

Before CEUS imaging analysis, the number, size, location (segment, intrahepatic or incisal margin), depth (from the skin surface to the center of the lesion), echogenicity (hypo-, iso-, hyper-, mixed-echoic), and liver background (degree of heterogeneity or beam attenuation: normal, moderate, severe), radiological cirrhosis and US visualization score were recorded. Visualization score was defined as follows: score A, no or minimal limitations (limitations unlikely to meaningfully affect sensitivity); score B, moderate limitations (limitations that may obscure small masses); and score C, severe limitations (limitations substantially lowering the sensitivity for focal liver lesions) [16].

To avoid recall bias, CT images were reviewed 2 weeks after the completion of CEUS evaluation, and so were MRI images. The reviewers also recorded the number, size, location, major imaging features and ancillary imaging features of the nodules.

### 2.4. Reference Standard

All malignant nodules included in this study were pathologically diagnosed through biopsy or surgical resection. For benign lesions, the reference standard was diagnosed by either histopathologic confirmation or size stability or regression at subsequent imaging for more than one year of follow-up. Liver cirrhosis was diagnosed through imaging (US/CT/MRI) of an irregular and nodular or shrunken liver together with impaired liver synthetic function (evidence of laboratory tests, portal hypertension, and splenomegaly) [17].

### 2.5. Statistical Analysis

All statistical analysis was performed using SPSS software (version 21.0). Descriptive analysis is reported as rates or percentages and absolute numbers. Continuous variables are expressed as medians and ranges. Comparisons between different groups were evaluated by using Student’s t-test for quantitative data and the chi-squared test or Fisher’s exact test for qualitative data. Inter-reader agreement between two readers on LI-RADS category is expressed as linear-weighted kappa coefficients with 95% confidence intervals (95% CIs). Kappa results were qualitatively stratified by score (κ = 0.81–1.00, almost perfect agreement; κ = 0.61–0.80, substantial agreement; κ = 0.41–0.60, moderate agreement; κ = 0.21–0.40, fair agreement; κ ≤ 0.20, slight agreement). The McNemar test or chi-squared test was performed to compare the sensitivity, specificity, accuracy, positive predictive value (PPV) and negative predictive value (NPV) of CEUS LI-RADS and CT/MRI LI-RADS considering different criteria (LR-5, LR-5/M and LR-4/5/M) as positive findings for recurrent HCC diagnosis. The optimal diagnostic algorithms for CEUS, CT and MRI were set based on receiver-operating characteristic (ROC) curve analysis to yield the largest Youden index. A two-sided *p*-value less than 0.05 indicates statistical significance.

## 3. Results

### 3.1. Patient Characteristics

A total of 303 patients (median age, 56 years; range, 18–90 years) with 421 lesions (median size, 17 mm; range, 5–96 mm) were eligible for this study. Among the 421 lesions, 218 were recurrent HCC and 203 were benign lesions. No non-HCC malignancy was found in these patients. In sum, 272 patients with 377 lesions underwent both CEUS and CT examinations, while 70 patients with 91 lesions underwent both CEUS and MRI examinations. Among these patients, 39 patients with 47 lesions underwent CEUS, CT and MRI. A total of 65 (15.4%) nodules were confirmed by biopsy 170 (40.4%) nodules were confirmed by surgical pathology, and 186 (44.2%) were confirmed by one-year follow-up. The demographic and clinical characteristics of the patients, including age, sex, nodule size, image modality and reference standard, are listed in Table 1.

### 3.2. CEUS LI-RADS Features between Different Nodule Characteristics and Liver Background

A total of 218 of the 421 (51.8%) nodules were recurrent HCC. No significant difference was found in rim enhancement regardless of nodule size, depth, location or liver background such as cirrhosis and visualization score (all *p* ≥ 0.05). Nodule size, depth, location and liver cirrhosis did not influence APHE, whereas visualization score affected APHE. APHE was more frequently presented on visualization scores A and B (96.3%, 183/190) than on visualization score C (82.1%, 23/28) (*p* = 0.010). The presence of the washout feature did not differ in nodule size, location, liver cirrhosis or visualization score, while nodule depth had a significant impact on the presence of washout. Washout was found to be significantly less in lesions of depth ≥ 8 cm (89.4%, 42/47) than < 8 cm (97.1%, 166/171) (*p* = 0.040) (Table 2).

### 3.3. Comparison between CEUS and CT/MRI on LI-RADS Major Features for Recurrent HCC

**Figure 2:**
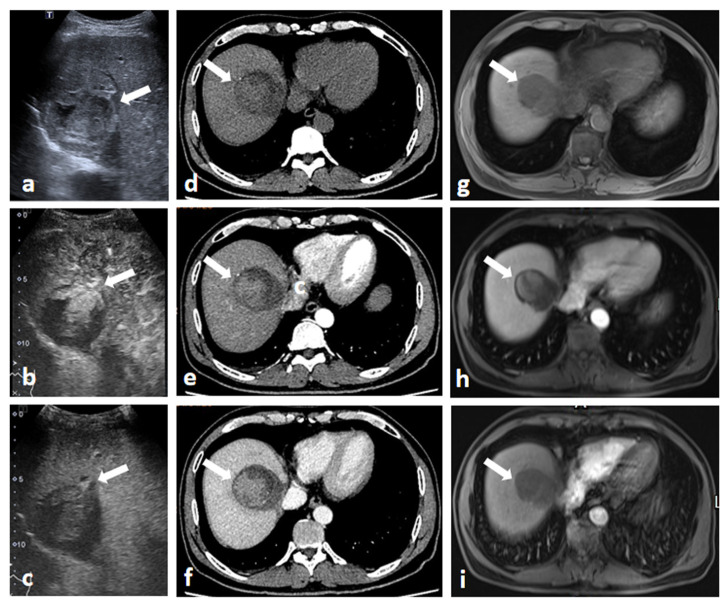
Images of a 54-year-old man with hepatocellular carcinoma treated with surgical resection after 3 months. The patient was confirmed to have no residual tumor during a one-month follow-up via contrast-enhanced CT. US image (**a**) showed a 5.6 cm hyperechoic lesion (white arrow) in segment 8 near the incisal margin. CEUS images demonstrated hyperenhancement on arterial phase (**b**) and washout on the late phase (**c**) which was designated LR-5 according to CEUS LI-RADS (white arrows). On axial unenhanced CT image (**d**), a hypoattenuated lesion (white arrow) was detected in segment 8 near the incisal margin. No hyperenhancement (white arrow) was detected on the arterial phase image (**e**), but hypoenhancement (white arrow) was seen on the portal venous phase (**f**). Axial T1-weighted unenhanced MR image (**g**) revealed an irregular hypointensity lesion located in segment 8 near the incisal margin. On the arterial phase (**h**) and portal venous phase (**i**), the mass remained hypoenhanced (white arrows). The lesion was confirmed to be recurrent HCC by histopathology.

Among 185 recurrent HCC, CEUS detected more APHE and washout (94.6%, 175/185 and 95.1%, 176/185, respectively) than CT (73.0%, 135/185 and 76.2%, 141/185) (*p* = 0.000), regardless of the size of lesions. Among 61 recurrent HCC, CEUS also detected more APHE (95.1%, 58/61) than MRI (80.3%, 49/61) (*p* = 0.025) and slightly more washout (95.1%, 58/61) than MRI (83.6%, 51/61), without significant difference (*p* > 0.05) (Figure 2).

No significant difference was observed in detecting rim enhancement between CEUS and CT/MRI (*p* > 0.05).

Threshold growth was found at 10.8% (20/185) on CT and 11.5% (7/61) on MRI, while enhancing capsule was found at 5.4% (10/185) on CT and 6.6% (4/61) on MRI (Table 3 and Table 4).

### 3.4. Optimal Diagnosis Algorithms on CEUS, CT and MRI for Diagnosing Recurrent HCC

ROC curve analysis demonstrated that using LR-4/5/M on CEUS, LR-4/5/M on CT and LR-5/M on MRI as positive criteria yielded the largest Youden index. The sensitivity, specificity, accuracy, PPV and NPV were 99.1% (216/218), 93.6% (190/203), 96.4% (406/421), 94.3% (216/229) and 99.0% (190/192) for CEUS LI-RADS, 90.3% (167/185), 90.1% (173/192), 90.2% (340/377), 89.8% (167/186) and 90.6% (173/191) for CT LI-RADS and 85.2% (52/61), 93.3% (28/30), 87.9% (80/91), 96.3% (52/54) and 75.7% (28/37) for MRI LI-RADS. The area under the curve (AUC) for recurrent HCC diagnosis was 0.981 (95%CI: 0.963–0.992) on CEUS, 0.958 (95%CI: 0.932–0.976) on CT and 0.933 (95%CI: 0.860–0.974) on MRI (Table 5).

### 3.5. Diagnostic Performance of Different Algorithms on CEUS and CT LI-RADS

With their optimal diagnosis algorithms, the diagnostic performance of CEUS LI-RADS using LR-4/5/M criteria outperformed CT LI-RADS using LR-4/5/M criteria for recurrent HCC diagnosis. The AUCs were 0.981 (95%CI: 0.962–0.992) on CEUS LI-RADS and 0.958 (95%CI: 0.932–0.976) on CT LI-RADS (*p* = 0.024). The diagnostic performance of different algorithms on CEUS and CT LI-RADS and their comparison are detailed in Table 6, Supplementary Material S1 and Table S1.

### 3.6. Diagnostic Performance of Different Algorithms on CEUS and MRI LI-RADS

With their optimal diagnosis algorithms, the diagnostic performance of CEUS LI-RADS using LR-4/5/M criteria was comparable to that of MRI LI-RADS using LR-5/M criteria for recurrent HCC, with AUC 0.952 (95%CI: 0.886–0.986) for CEUS LI-RADS and 0.933 (95%CI: 0.860–0.974) for MRI LI-RADS respectively (*p* > 0.05). The diagnostic performance of different algorithms on CEUS and MRI LI-RADS and their comparison are detailed in Table 6, Supplementary Material S1 and Table S2.

**Table 6:** Comparison of diagnostic performance of CEUS, CT and MRI using their optimal diagnostic for recurrent HCC.

	TP *	TN *	FP *	FN *	Sensitivity	Specificity	Accuracy	PPV	NPV	AUC
*n* = 377										
CEUS (LR-4/5/M)	183	180	12	2	98.9 (183/185) [96.2, 99.9]	93.8 (180/192) [89.3, 96.7]	96.3 (363/377) [94.4, 98.2]	93.8 (183/195) [89.5, 96.8]	98.9 (180/182) [96.1, 99.9]	0.981 [0.962, 0.992]
CT (LR-4/5/M)	167	173	19	18	90.3 (167/185) [85.1, 94.1]	90.1 (173/192) [85.0, 93.9]	90.2 (340/377) [87.2, 93.2]	89.8 (167/186) [84.5, 93.7]	90.6 (173/191) [85.5, 94.3]	0.958 [0.932, 0.976]
*p* value					0.000	0.189	0.000	0.189	0.000	0.024
*n* = 91										
CEUS (LR-4/5/M)	60	26	4	1	98.4 (60/61) [91.2, 100]	86.7 (26/30) [69.3, 96.2]	94.5 (86/91) [89.8, 99.2]	93.8 (60/64) [84.8, 98.3]	96.3 (26/27) [81.0, 99.9]	0.952 [0.886, 0.986]
MRI (LR-5/M)	52	28	2	9	85.2 (52/61) [73.8, 93.0]	93.3 (28/30) [77.9, 99.2]	87.9 (80/91) [81.2, 94.6]	96.3 (52/54) [87.3, 99.6]	75.7 (28/37) [58.8, 88.2]	0.933 [0.860, 0.974]
*p* value					0.008	0.500	0.109	0.686	0.035	0.598

Note: A total of 272 patients with 377 lesions underwent both CT and CEUS, and 70 patients with 91 lesions underwent both MRI and CEUS. Unless otherwise indicated, data are percentages and those in parentheses are the numerator/denominator. Data in brackets are 95% confidence intervals. * Data are numbers of cases. HCC: hepatocellular carcinoma, LI-RADS: Liver Imaging Reporting and Data System, CEUS: contrast-enhanced ultrasonography, CT: computer tomography, MRI: magnetic resonance imaging, AUC: area under the curve, PPV: positive predictive value, NPV: negative predictive value, TP: true positive, TN: true negative, FP: false positive, FN: false negative.

### 3.7. Comparison of Diagnostic Performance of CEUS, CT and MRI LI-RADS

In sum, 39 patients with 47 lesions (47/421, 11.2%) underwent all CEUS, CT and MRI examinations, with 28 (28/47, 59.6%) malignant lesions and 19 (19/47, 40.4%) benign lesions. The diagnostic performance of different algorithms on CEUS, CT and MRI LI-RADS is presented in Table S3 and the strengths and drawbacks of each technique are presented in Table S4.

### 3.8. Inter-Reader Agreement on LI-RADS Categories

The inter-reader agreement of LI-RADS categories on CEUS, CT and MRI is detailed in the Supplementary Materials S1 and Table S5.

### 3.9. The Detection Rate of Recurrent HCC on CEUS, CT and MRI

During the image review and pathology confirmation process, we found that another 12 (12/230, 5.2%) recurrent HCCs were detected on CT or MRI and missed on CEUS. The locations of these lesions were hepatic segment 2 or 3 near the stomach, hepatic segment 7 or 8 adjacent to the dome of the diaphragm, and the lower part of hepatic segment 6, which was easily shadowed by bowel gas and hepatic segment 1. The detection rate of recurrent HCC in visualization scores A, B and C was 98.1% (52/53), 95.8% (138/144) and 84.8% (28/33), respectively. The detection rate of recurrent HCC in visualization score C was lower than that in visualization scores A and B (*p* = 0.017).

**Figure 3:**
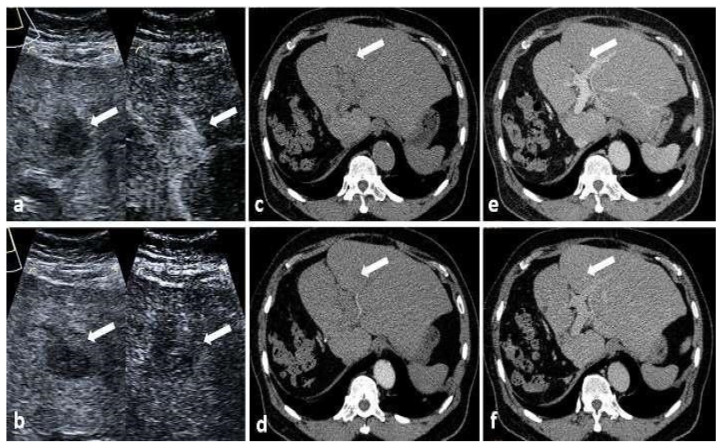
Images of a 72-year-old man with HCC treated with surgical resection for nearly 3 years. (**a**) US image showed a 1.9 cm hypoechoic nodule (white arrow) located in hepatic segment 2. The lesion demonstrated hyperenhancement on the arterial phase and hypoenhancement on the late phase (**b**) (white arrow). On axial CT images, the lesion was not detected on unenhanced phase (**c**), arterial phase (**d**), portal venous phase (**e**) or delay phase (**f**) (white arrows). A biopsy was performed and the lesion was confirmed as recurrent HCC by histopathology.

There was only one (1/185, 0.5%) lesion missed on CT, which was invisible in all phases, but delineated on CEUS (Figure 3). No lesion was missed on MRI. The detection rates of CEUS, CT and MRI was 94.8% (218/230), 99.5% (193/194) and 100% (66/66), respectively. The detection rate of recurrent HCC on CEUS (94.8%, 218/230) was lower than that on CT/MRI (99.6%, 259/260) (*p* = 0.001).

## 4. Discussion

This is the first comparative study to evaluate the diagnostic performance of CEUS LI-RADS and CT/MRI LI-RADS in recurrent HCC, and revealed that CEUS, CT and MRI play complementary roles in the characterization and detection of recurrent HCC. CEUS displayed a relatively lower detection rate while yielding better diagnostic performance than CT/MRI.

In lesion characterization, using LR-5 as a diagnostic criterion for CEUS, CT and MRI demonstrated a relatively low diagnostic performance, indicating that the LR-5 criterion is not optimal in recurrent HCC diagnosis. In this study, no non-HCC malignancy was detected and all CEUS LR-M nodules in the malignant group were pathologically confirmed as recurrent HCC, suggesting the inclusion of CEUS LR-M to reform new diagnostic criteria for recurrent HCC diagnosis may be possible. To achieve better diagnostic performance, refining a diagnostic algorithm for recurrent HCC is needed. ROC curve analysis revealed that LR-4/5/M criteria were optimal for diagnosing HCC recurrence for CEUS and CT and LR-5/M criteria for MRI, all of which demonstrated the highest diagnostic accuracy.

No studies have ever evaluated or compared the diagnostic performance of CEUS and CT/MRI LI-RADS in recurrent HCC. Our study suggests that CEUS has its superiority in recurrent HCC characterization. The excellent diagnostic performance of CEUS was highly attributable to its accurate detection of LI-RADS major features for recurrent HCC. In this study, CEUS offered higher sensitivity for detecting APHE and washout for recurrent HCC regardless of lesion size compared with CT and higher sensitivity for APHE detection compared with MRI. We assumed that several possible reasons may contribute to this phenomenon. First, CEUS, with its real-time, dynamic, and continuous characteristics, can capture the wash-in and wash-out of the contrast media within the nodule, other than that acquired at static fixed time points on CT/MRI [14]. Second, CEUS revealed precise and obvious washout of the nodule with its pure blood pool contrast agent, ruling out interstitial contrast agent leakage demonstrated on CT [14]. Third, repeated treatments may impair the liver and worsen cirrhosis, resulting in reduced enhancement of the lesion on CT/MRI [18,19].

In evaluating factors that influence recurrent HCC characterization on CEUS, we found lesion depth ≥ 8 cm may hinder the detection of washout, whereas visualization score C influenced the observation of APHE. We hypothesized that deeper hepatic lesions may be obscured by the attenuation of the US beam with depth-dependent loss of contrast, which reduced the detection of washout features [20,21,22]. No study had ever correlated visualization score with CEUS major features for recurrent HCC. Therefore, in cases of indeterminate nodules manifesting atypical enhancement patterns, particularly those of depth ≥ 8 cm or visualization score C, the utilization of CT/MRI may be instrumental in facilitating a correct diagnosis.

In recurrent HCC detection, the applicability of CEUS may be questionable for its limitation of visualizing the whole liver. Therefore, we further analyzed the location of the missed lesions and the detection rate in different visualization scores on CEUS. In the present study, 12 lesions located in the blind spots of the US were missed by CEUS, which were easily shadowed by gas from lung or hollow viscera. The detection rate of recurrent HCC in visualization score C was the lowest, which may be attributed to poor sonic windows and coarse echotexture in the liver background. These results agree with previous studies suggesting lesions located in the blind spots of the US and inadequate visualization limited the capacity of HCC detection [23,24,25]. These missed lesions on CEUS were all detected by CT/MRI, indicating CT/MRI has an advantage over CEUS in lesion detection.

In clinical practice, CT and MRI are the preferred imaging modalities for recurrent HCC on both detection and characterization, while CEUS is not accepted as the first-line modality [26,27]. However, this study revealed that for ultrasound-detected lesions, CEUS allowed for better detection of LI-RADS major features compared with CT and MRI, resulting in excellent diagnostic performance for recurrent HCC. CEUS can resolve equivocal CT/MRI observations, which facilitates a correct diagnosis and activate subsequent retreatment. Nevertheless, the limitation of CEUS in lesion detection cannot be neglected. In this study, visualization score C and lesions located on the blind spots of the US would hinder the ability to detect recurrent HCC. Among the three modalities, CT/MRI demonstrated a higher detection rate compared to CEUS (*p* = 0.001). Therefore, CT/MRI may serve as a preferred surveillance modality for recurrent HCC, while CEUS can be employed as an excellent diagnostic tool for observation when CT/MRI results are inconclusive.

Our study is subject to certain limitations. Firstly, the utilization of data from a single institution may introduce inherent bias. Notably, there is an inclusion bias in this study as it exclusively encompasses patients presenting with a new lesion on CEUS. Secondly, the diagnostic reference standard for recurrent HCC in this investigation adhered to a stringent pathological diagnosis, potentially resulting in a relatively limited sample size and the presence of selection biases. Thirdly, the statistical analyses were conducted on a per-lesion basis, which may overlook potential interactions among multiple additional focal lesions when assigning LI-RADS categories. Moreover, this study lacks a cost-effectiveness analysis to compare the three modalities for diagnosing recurrent HCC.

## 5. Conclusions

In conclusion, CEUS and CT/MRI play complementary roles in the detection and characterization of recurrent HCC. CT/MRI may be used as a surveillance modality, whereas CEUS can be an excellent diagnostic modality using LR-4/5/M as the diagnosis criterion, which outperformed CT while being comparable to MRI.

## Figures and Tables

**Table 1:** Characteristics of patients.

Characteristic	Recurrent HCC	Benign Lesions	*p*-Value
**Per-patient**			
Total	174/303 (57.4)	147/303 (48.5)	0.034
Sex			0.467
Male	158/174 (90.8)	129/147 (87.8)	
Female	16/174 (9.2)	18/147 (12.2)	
Age, median ^§^	56 (18–90)	58 (18–84)	0.122
**Per-lesion**	218/421 (51.8)	203/421 (48.2)	
Size (mm), median ^§^	25.5 (8–96)	14.0 (5–42)	0.000
Image modality			
CEUS and CT	185/377 (49.1)	192/377 (50.9)	
CEUS and MRI	61/91 (67.0)	30/91 (33.0)	0.002
Reference standard			
Needle biopsy	55/218 (25.2)	10/203 (4.9)	0.000
Surgery	163/218 (74.8)	7/203 (3.4)	0.000
Clinically confirmed	0/218 (0)	186/203 (91.6)	0.000

Note: We included 18 patients who had recurrent HCC or benign lesions at different follow-up time points. Unless otherwise indicated, data are the numerator/denominator of patients and data in parentheses are percentages. ^§^ Data are median values and those in parentheses are range. Bold characters presents the following items are analyzed based on per-patient level and per-lesion level respectively. HCC: hepatocellular carcinoma, CEUS: contrast-enhanced ultrasonography, CT: computer tomography, MRI: magnetic resonance imaging.

**Table 2:** Comparison of CEUS LI-RADS features between different nodule characteristics and liver background for recurrent HCC (*n* = 218).

	APHE	Washout	Rim Enhancement
Size			
≥2 cm	93.4 (141/151)	96.7 (146/151)	2.6 (4/151)
<2 cm	97.0 (65/67)	92.5 (62/67)	1.5 (1/67)
Depth			
≥8 cm	95.7 (45/47)	89.4 (42/47) *	2.1 (1/47)
<8 cm	94.2 (161/171)	97.1 (166/171) *	2.3 (4/171)
Location Incisal margin	93.5 (29/31)	100 (31/31)	3.2 (1/31)
Intrahepatic	94.7 (177/187)	94.7 (177/187)	2.1 (4/187)
Cirrhosis			
Cirrhosis	91.7 (66/72)	91.7 (66/72)	2.8 (2/72)
Non-cirrhosis	95.9 (140/146)	97.3 (142/146)	2.1 (3/146)
Visualization score			
A + B	96.3 (183/190) *	96.3 (183/190)	2.1 (4/190)
C	82.1 (23/28) *	89.3 (25/28)	3.6 (1/28)

Data are percentages and those in parentheses are the numerator/denominator of recurrent HCC. HCC: hepatocellular carcinoma, LI-RADS: Liver Imaging Reporting and Data System, CEUS: contrast-enhanced ultrasonography, MRI: magnetic resonance imaging, APHE: arterial phase hyperenhancement. * *p* < 0.05.

**Table 3:** Comparison between CEUS and CT on LI-RADS major features for recurrent HCC (*n* = 185).

	CEUS	CT	*p* Value
APHE	94.6 (175/185)	73.0 (135/185)	0.000
≥2 cm	93.5 (115/123)	73.2 (90/123)	0.000
<2 cm	96.8 (60/62)	72.6 (45/62)	0.000
Washout	95.1 (176/185)	76.2 (141/185)	0.000
≥2 cm	96.7 (119/123)	80.5 (99/123)	0.000
<2 cm	91.9 (57/62)	67.7 (42/62)	0.001
Rim enhancement	2.2 (4/185)	2.7 (5/185)	1.000
≥2 cm	2.4 (3/123)	4.1 (5/123)	0.722
<2 cm	1.6 (1/62)	0 (0/62)	1.000
Threshold growth	——	10.8 (20/185)	——
≥2 cm	——	12.2 (15/123)	——
<2 cm	——	8.1 (5/62)	——
Enhancing capsule	——	5.4 (10/185)	——
≥2 cm	——	8.1 (10/123)	——
<2 cm	——	0 (0/62)	——

Data are percentages and those in parentheses are the numerator/denominator of recurrent HCCs. HCC: hepatocellular carcinoma, LI-RADS: Liver Imaging Reporting and Data System, CEUS: contrast-enhanced ultrasonography, MRI: magnetic resonance imaging, APHE: arterial phase hyperenhancement.

**Table 4:** Comparison between CEUS and MRI on LI-RADS major features (*n* = 61).

	CEUS	MRI	*p* Value
APHE	95.1 (58/61)	80.3 (49/61)	0.025
≥2 cm	95.7 (44/46)	80.4 (37/46)	0.050
<2 cm	93.3 (14/15)	80.0 (12/15)	0.598
Washout	95.1 (58/61)	83.6 (51/61)	0.075
≥2 cm	97.8 (45/46)	84.8 (39/46)	0.059
<2 cm	86.7 (13/15)	80.0 (12/15)	1.000
Rim enhancement	1.6 (1/61)	8.2 (5/61)	0.207
≥2 cm	2.2 (1/46)	8.7 (4/46)	0.361
<2 cm	0 (0/15)	6.7 (1/15)	1.000
Threshold growth	——	11.5 (7/61)	——
≥2 cm	——	15.2 (7/46)	——
<2 cm	——	0 (0/15)	——
Enhancing capsule	——	6.6 (4/61)	——
≥2 cm	——	8.7 (4/46)	——
<2 cm	——	0 (0/15)	——

Data are percentages and those in parentheses are the numerator/denominator of recurrent HCCs. HCC: hepatocellular carcinoma, LI-RADS: Liver Imaging Reporting and Data System, CEUS: contrast-enhanced ultrasonography, MRI: magnetic resonance imaging, APHE: arterial phase hyperenhancement.

**Table 5:** Optimal algorithms on CEUS, CT and MRI for diagnosing recurrent HCC.

	TP *	TN *	FP *	FN *	Sensitivity	Specificity	Accuracy	PPV	NPV	AUC
CEUS (*n* = 421)										
LR-4/5/M	216	190	13	2	99.1 (216/218) [96.7, 99.9]	93.6 (190/203) [89.3, 96.6]	96.4 (406/421) [94.7, 98.2]	94.3 (216/229) [90.5, 96.9]	99.0 (190/192) [96.3, 99.9]	0.981 [0.963, 0.992]
CT (*n* = 377)										
LR-4/5/M	167	173	19	18	90.3 (167/185) [85.1, 94.1]	90.1 (173/192) [85.0, 93.9]	90.2 (340/377) [87.2, 93.2]	89.8 (167/186) [84.5, 93.7]	90.6 (173/191) [85.5, 94.3]	0.958 [0.932, 0.976]
MRI (*n* = 91)										
LR-5/M	52	28	2	9	85.2 (52/61) [73.8, 93.0]	93.3 (28/30) [77.9, 99.2]	87.9 (80/91) [81.2, 94.6]	96.3 (52/54) [87.3, 99.6]	75.7 (28/37) [58.8, 88.2]	0.933 [0.860, 0.974]

Note: A total of 303 patients with 421 lesions underwent CEUS, 272 patients with 377 lesions underwent both CT and CEUS, and 70 patients with 91 lesions underwent both MRI and CEUS. Among these patients, 39 patients with 47 lesions underwent CEUS, CT and MRI. Unless otherwise indicated, data are percentages and those in parentheses are the numerator/denominator. Data in brackets are 95% confidence intervals. * Data are numbers of cases. HCC: hepatocellular carcinoma, LI-RADS: Liver Imaging Reporting and Data System, CEUS: contrast-enhanced ultrasonography, CT: computer tomography, MRI: magnetic resonance imaging, AUC: area under the curve, PPV: positive predictive value, NPV: negative predictive value, TP: true positive, TN: true negative, FP: false positive, FN: false negative.

## Data Availability

The ultrasound and clinical data are publicly unavailable due to patient confidentiality reasons and privacy protection.

## Supplementary Materials

The following supporting information can be downloaded at: https://www.mdpi.com/article/10.3390/cancers15245743/s1. Table S1: Comparison of diagnostic performance of CEUS and CT using LR-5, LR-5/M and LR-4/5/M as diagnostic criteria for recurrent HCC; Table S2: Comparison of diagnostic performance of CEUS and MRI using LR-5, LR-5/M and LR-4/5/M as diagnostic criteria for recurrent HCC; Table S3: Comparison of diagnostic performance of CEUS, CT and MRI using LR-5, LR-5/M and LR-4/5/M as diagnostic criteria for recurrent HCC; Table S4: The strengths and drawbacks of CEUS and CT/ MRI in diagnosing recurrent HCC; Table S5: The inter-reader agreement for Liver Imaging Reporting and Data System categories on CEUS, CT and MRI.

## Abbreviation

HCC	hepatocellular carcinoma
LI-RADS	Liver Imaging Reporting and Data System
ACR	American College of Radiology
CEUS	contrast-enhanced ultrasonography
CT	computer tomography
MRI	magnetic resonance imaging
APHE	arterial phase hyperenhancement
PPV	positive predictive value
NPV	negative predictive value
PVP	portal venous phase
TP	transitional phase
HBP	hepatobiliary phase
AP	arterial phase
AUC	area under the curve
US	ultrasound
