# Supplementary material for: Complementary Role of CEUS and CT/MR LI-RADS for Diagnosis of Recurrent HCC
Source: Cancers (Basel). 2023 Dec 7;15(24):5743. doi: 10.3390/cancers15245743 (PMC10741803; doi:10.3390/cancers15245743)
Supplement: Supplementary file 1 [file cancers-15-05743-s001.zip › cancers-2695681-supplementary.pdf]

Supplementary Material S1

Image archiving and scanning parameters of CT and MRI

CEUS examination

The majority of US and CEUS examinations were performed by either an Aplio 500 machine (Toshiba Medical Systems, Tokyo, Japan) with a 375BT convex transducer (frequency range, 1.9-6.0 MHz) or an Aixplorer Ultrasound system (SuperSonic Imagine, Aix-en-Provence, France) equipped with an SC6-1 convex probe (frequency range, 1.0-6.0 MHz). The examination for this study was highly standardized and was separately performed by two expert radiologists (W.W. and M.X.L) with at least 15 years of experience in liver images. On baseline US, the number, size, location, echogenicity of lesions, and liver background were recorded.

For CEUS examinations, low-mechanical index and real-time subtraction technique with Dual-screen mode were set when evaluating the target liver nodule. CEUS images of the target lesion and surrounding liver parenchyma were continuously recorded for the first 90 s without any change in machine settings after a bolus injection of 2.4 mL of SonoVue (Bracco, Milan, Italy) in the antecubital vein followed by a 5 mL saline flush. After 90 seconds, the lesion was intermittently scanned and stored for at least 5 minutes to evaluate its washout features. All image data were stored for further evaluation.

CT examination

Contrast-enhanced CT was performed with multidetector CT (MDCT) scanners: a 64-detector row (Aquilion CXL, Toshiba Medical System, Tokyo, Japan) or a 320-detector row CT machine (Aquilion One, Toshiba Medical System, Tokyo, Japan). All CT examinations included an unenhanced phase and three vascular phases covering the upper abdomen. The scanning parameters were as follows: tube voltage, 120-kVp; tube current, 250 mA; and section thickness slice, 1 mm. After a routine unenhanced scan, a dose of 1.5 mL/kg of warmed contrast medium (Ultravist, Bayer, Germany) was automatically injected with a mechanical power injector (P3T abdomen module, Medrad Inc.) at a rate of 3.0 mL/s through a 20-G catheter into an antecubital vein. Hepatic arterial phase (AP), portal venous phase (PVP), and equilibrium phase images were obtained at 35 s, 65 s and 180 s, respectively from the start of the contrast material injection.

MRI examination

MRI images were scanned using a 3.0 T MR system (SIGNA Pioneer, GE Healthcare, WI, USA) or 3.0 T MR system (Magnetom Verio, Siemens Healthineers, Erlangen, Germany) in a supine position with an eight-channel phased-array torso coil centered over the abdomen. The scanning scale covered from the top to the lower edge of the liver. Routine MRI sequences included were as follows: T2-weighted, diffusion-weighted, in- and out-of-phase, unenhanced T1-weighted, dynamic, and hepatobiliary phase (HBP) sequences. The contrast agent was automatically injected into an antecubital vein via a power injection device at a rate of 1.0 mL/s for gadoxetate disodium (Primovist; Bayer Healthcare) for a total dose of 0.025 mmol/kg body weight, or at a rate of 2 ml/s for a dose of 0.1 mmol/kg body weight for the extracellular gadolinium contrast agent, followed by a 20 mL saline flush. According to CT/MRI LI-RADS, The timing for the AP was 15–30 s after the contrast agent arrived at the pulmonary artery, with the PVP, transitional phase (TP) and HBP at 50–80 s, 3 min and 20 min, respectively.

Diagnostic performance of different algorithms on CEUS and CT LI-RADS

When considering LR-5 as a positive finding, the sensitivity, specificity, accuracy, PPV and NPV of CEUS LI-RADS and CT LI-RADS were 62.7% (116/185) vs. 64.9% (120/185), 100.0% (192/192) vs. 99.0% (190/192), 81.7% (308/377) vs. 82.2 % (310/377), 100.0% (116/116) vs. 98.4% (120/122) and 73.6% (192/261) vs. 74.5% (190/255), respectively. No statistically significant difference of diagnostic performance was found between CEUS and CT (all $P > 0.05$).

When considering LR-5/M as a positive finding, the sensitivity, specificity, accuracy, PPV and NPV of CEUS LI-RADS and CT LI-RADS were 93.5% (173/185) vs. 67.6% (125/185), 98.4% (189/192) vs. 99.0 % (190/192), 96.0% (362/377) vs. 83.6% (315/377) and 98.3% (173/176) vs 98.4% (125/127) and 94.0 % (189/201) vs.76.0% (190/250), respectively. The sensitivity, accuracy and NPV of CEUS were higher than CT ($P = 0.000$, $P = 0.000$ and $P = 0.000$, respectively). In the 60 false negative cases of CT using LR-5/M as diagnostic criteria, 88.3% (53/60) were accurately characterized by CEUS, which accounted for 28.6% (53/185) of all recurrent HCC.

When considering LR-4/5/M as a positive finding, the sensitivity, specificity, accuracy, PPV and NPV of CEUS LI-RADS and CT LI-RADS were 98.9% (183/185) vs. 90.3% (167/185), 93.8% (180/192) vs. 90.1% (173/192), 96.3% (363/377) vs. 90.2% (340/377) and 93.8% (183/195) vs 89.8% (167/186) and 98.9 % (180/182) vs. 90.6% (173/191), respectively. CEUS demonstrated higher sensitivity, accuracy and NPV than CT ($P = 0.000$, $P = 0.000$ and $P = 0.000$, respectively) (Table 5). In

the 18 false negative cases of CT using LR-4/5/M as diagnostic criteria, 100% (18/18) were accurately characterized by CEUS, which accounted for 9.7% (18/185) of all recurrent HCC (Table S1).

Diagnostic performance of different algorithms on CEUS and MRI LI-RADS

When using LR-5 as a positive criteria, the sensitivity, specificity, accuracy, PPV and NPV of CEUS LI-RADS and MRI LI-RADS were 65.6% (40/61) vs. 77.0% (47/61), 96.7% (29/30) vs.93.3% (28/30), 75.8% (69/91) vs. 82.4% (75/91), 97.6% (40/41) vs 95.9% (47/49) and 58.0% (29/50) vs. 66.7% (28/42), respectively. No statistically significant difference of diagnostic performance was found between CEUS and MRI (all $P > 0.05$).

When using LR-5/M as a positive criterion, the sensitivity, specificity, accuracy, PPV and NPV of CEUS LI-RADS and MRI LI-RADS were 96.7% (59/61) vs. 85.2% (52/61), 93.3% (28/30) vs. 93.3% (28/30), 95.6% (87/91) vs. 87.9% (80/91), 96.7% (59/61) vs 96.3% (52/54) and 93.3% (28/30) vs. 75.7% (28/37), respectively. The sensitivity of CEUS was higher than MRI ($P = 0.039$). In the 9 false-negative cases of MRI using LR-5/M as diagnostic criteria, 88.9% (8/9) were accurately characterized by CEUS, which account for 4.3% (8/185) of all recurrent HCC.

When using LR-4/5/M as a positive criterion, the sensitivity, specificity, accuracy, PPV and NPV of CEUS LI-RADS and MRI LI-RADS were 98.4% (60/61) vs. 100.0% (61/61), 86.7% (26/30) vs. 50.0% (15/30), 94.5% (86/91) vs. 83.5% (76/91), 93.8% (60/64) vs 80.3% (61/76) and 96.3% (26/27) vs. 100.0% (15/15), respectively. CEUS achieved higher specificity, accuracy and PPV than MRI ($P = 0.003$, $P = 0.013$ and $P = 0.025$, respectively) (Table 6). In the 15 false positive cases of MRI using LR-4/5/M as diagnostic criteria, 80.0% (12/15) were accurately characterized by CEUS, which accounted for 40% (12/30) of all benign lesions (Table S2).

Inter-reader agreement on LI-RADS categories

The inter-reader agreement was almost perfect with κ values of 0.893 (95%CI 0.862, 0.924) for CEUS LI-RADS categories, almost perfect with κ values of 0.873 (95%CI 0.839, 0.907) for CT LI-RADS categories and substantial with κ values of 0.784 (95%CI 0.663, 0.905) for MRI LI-RADS categories (Table S4)

Table S1 Comparison of diagnostic performance of CEUS and CT using LR-5, LR-5/M and LR-4/5/M as diagnostic criteria for recurrent HCC

	TP*	TN*	FP*	FN*	Sensitivity	Specificity	Accuracy	PPV	NPV	AUC
LR-5										
CEUS	116	192	0	69	62.7(116/185)	100(192/192)	81.7(308/377)	100(116/116)	73.6(192/261)	0.981
					[55.3, 69.7]	[98.1, 100]	[77.8, 85.6]	[96.9, 100]	[67.8, 78.8]	[0.962,0.992]
CT	120	190	2	65	64.9(120/185)	99.0(190/192)	82.2(310/377)	98.4(120/122)	74.5(190/255)	0.958
					[57.5, 71.7]	[96.3, 99.9]	[78.4, 86.1]	[94.2, 99.8]	[68.7, 79.7]	[0.932,0.976]
P value					0.731	0.500	0.910	0.498	0.841	0.024
LR-5/M										
CEUS	173	189	3	12	93.5(173/185)	98.4(189/192)	96.0(362/377)	98.3(173/176)	94.0(189/201)	0.981
					[88.9, 96.6]	[95.5, 99.7]	[94.0, 98.0]	[95.1, 99.7]	[89.8, 96.9]	[0.962,0.992]
CT	125	190	2	60	67.6(125/185)	99.0(190/192)	83.6(315/377)	98.4(125/127)	76.0(190/250)	0.958
					[60.3, 74.3]	[96.3, 99.9]	[79.8, 87.3]	[94.4, 99.8]	[70.2, 81.2]	[0.932,0.976]
P value					0.000	1.000	0.000	1.000	0.000	0.024
LR-4/5/M										
CEUS	183	180	12	2	98.9(183/185)	93.8(180/192)	96.3(363/377)	93.8(183/195)	98.9(180/182)	0.981
					[96.2, 99.9]	[89.3, 96.7]	[94.4, 98.2]	[89.5, 96.8]	[96.1, 99.9]	[0.962,0.992]
CT	167	173	19	18	90.3(167/185)	90.1(173/192)	90.2(340/377)	89.8(167/186)	90.6(173/191)	0.958
					[85.1, 94.1]	[85.0, 93.9]	[87.2, 93.2]	[84.5, 93.7]	[85.5, 94.3]	[0.932,0.976]
P value					0.000	0.189	0.000	0.189	0.000	0.024

Unless otherwise indicated, Data are percentages and data in parentheses are the numerator/denominator, data in brackets are 95% confidence intervals

* Data are numbers of cases.

HCC hepatocellular carcinoma, LI-RADS Liver Imaging Reporting and Data System, CEUS contrast-enhanced ultrasonography,

CT computer tomography, APHE arterial phase hyperenhancement, AUC area under the curve, PPV positive predictive value, NPV negative predictive value, TP true positive, TN true negative, FP false positive, FN false negative

Table S2 Comparison of diagnostic performance of CEUS and MRI using LR-5, LR-5/M and LR-4/5/M as diagnostic criteria for recurrent HCC

	TP*	TN*	FP*	FN*	Sensitivity	Specificity	Accuracy	PPV	NPV	AUC
LR-5										
CEUS	40	29	1	21	65.6(40/61)	96.7(29/30)	75.8(69/91)	97.6(40/41)	58.0(29/50)	0.952
					[52.3, 77.3]	[82.8, 99.9]	[67.0, 84.6]	[87.1, 99.9]	[43.2, 71.8]	[0.886,0.986]
MRI	47	28	2	14	77.0(47/61)	93.3(28/30)	82.4(75/91)	95.9(47/49)	66.7(28/42)	0.933
					[64.5, 86.9]	[77.9, 99.2]	[74.6, 90.2]	[86.0, 99.5]	[50.5, 80.4]	[0.860,0.974]
P value					0.167	1.000	0.263	1.000	0.518	0.598
LR-5/M										
CEUS	59	28	2	2	96.7(59/61)	93.3(28/30)	95.6(87/91)	96.7(59/61)	93.3(28/30)	0.952
					[88.7, 99.6]	[77.9, 99.2]	[91.4, 99.8]	[88.7, 99.6]	[77.9, 99.2]	[0.886,0.986]
MRI	52	28	2	9	85.2(52/61)	93.3(28/30)	87.9(80/91)	96.3(52/54)	75.7(28/37)	0.933
					[73.8, 93.0]	[77.9, 99.2]	[81.2, 94.6]	[87.3, 99.6]	[58.8, 88.2]	[0.860,0.974]
P value					0.039	1.000	0.065	1.000	0.095	0.598
LR-4/5/M										
CEUS	60	26	4	1	98.4(60/61)	86.7(26/30)	94.5(86/91)	93.8(60/64)	96.3(26/27)	0.952
					[91.2, 100]	[69.3, 96.2]	[89.8, 99.2]	[84.8, 98.3]	[81.0, 99.9]	[0.886,0.986]
MRI	61	15	15	0	100(61/61)	50(15/30)	83.5(76/91)	80.3(61/76)	100(15/15)	0.933
					[94.1, 100]	[31.3, 68.7]	[75.9, 91.1]	[69.5, 88.5]	[78.2, 100]	[0.860,0.974]
P value					1.000	0.003	0.013	0.025	1.000	0.598

Unless otherwise indicated, Data are percentages and data in parentheses are the numerator/denominator, data in brackets are 95% confidence intervals

* Data are numbers of cases.

HCC hepatocellular carcinoma, LI-RADS Liver Imaging Reporting and Data System, CEUS contrast-enhanced ultrasonography,

MRI magnetic resonance imaging, APHE arterial phase hyperenhancement, AUC area under the curve, PPV positive predictive value, NPV negative predictive value, TP true positive, TN true negative, FP false positive, FN false negative

Table S3 Comparison of diagnostic performance of CEUS, CT and MRI using LR-5, LR-5/M and LR-4/5/M as diagnostic criteria for recurrent HCC

	TP*	TN*	FP*	FN*	Sensitivity	Specificity	Accuracy	PPV	NPV	AUC
LR-5										
CEUS	18	19	0	10	64.3(18/28)	100(19/19)	78.7(37/47)	100(18/18)	65.5(19/29)	0.937
					[44.1, 81.4]	[82.4, 100]	[67.0, 90.4]	[81.5, 100]	[45.7, 82.1]	[0.826, 0.987]
CT	12	19	0	16	42.9(12/28)	100(19/19)	66.0(31/47)	100(12/12)	54.3(19/35)	0.914
					[24.5, 62.8]	[82.4, 100]	[52.4, 79.5]	[73.5, 100]	[36.7, 71.2]	[0.794, 0.976]
MRI	22	18	1	6	78.6(22/28)	94.7(18/19)	85.1(40/47)	95.7(22/23)	75.0(18/24)	0.948
					[59.1, 91.7]	[74.0, 99.9]	[74.9, 95.3]	[78.1, 99.9]	[53.3, 90.2]	[0.841, 0.992]
P value					0.022	0.361	0.083	0.208	0.258	——
LR-5/M										
CEUS	26	18	1	2	92.9(26/28)	94.7(18/19)	93.6(44/47)	96.3(26/27)	90.0(18/20)	0.937
					[76.5, 99.1]	[74.0, 99.9]	[86.7, 100]	[81.0, 99.9]	[63.8, 98.8]	[0.826, 0.987]
CT	12	19	0	16	42.9(12/28)	100(19/19)	66.0(31/47)	100(12/12)	54.3(19/35)	0.914
					[24.5, 62.8]	[82.4, 100]	[52.4, 79.5]	[73.5, 100]	[36.7, 71.2]	[0.794, 0.976]
MRI	25	18	1	3	89.3(25/28)	94.7(18/19)	91.5(43/47)	96.2(25/26)	85.7(18/21)	0.948
					[71.8, 97.7]	[74.0, 99.9]	[83.5, 99.5]	[80.4, 99.9]	[63.7, 97.0]	[0.841, 0.992]
					0.000	0.596	0.000	0.732	0.005	——
LR-4/5/M										
CEUS	27	16	3	1	96.4(27/28)	84.2(16/19)	91.5(43/47)	90.0(27/30)	94.1(16/17)	0.937
					[81.7, 99.9]	[60.4, 96.6]	[83.5, 99.5]	[73.5, 97.9]	[71.3, 99.9]	[0.826, 0.987]
CT	24	16	3	4	85.7(24/28)	84.2(16/19)	85.1(40/47)	88.9(24/27)	80.0 (16/20)	0.914
					[67.3, 96.0]	[60.4, 96.6]	[74.9, 95.3]	[70.8, 97.7]	[56.3, 94.3]	[0.794, 0.976]
MRI	28	9	10	0	100(28/28)	47.4(9/19)	78.7(37/47)	73.7(28/38)	100(9/9)	0.948
					[87.7, 100]	[24.5, 71.1]	[67.0, 90.4]	[56.9, 86.6]	[66.4, 100]	[0.841, 0.992]
P value					0.063	0.002	0.152	0.034	0.241	——

Unless otherwise indicated, Data are percentages and data in parentheses are the numerator/denominator

Data in brackets are 95% confidence intervals

* Data are numbers of cases

HCC hepatocellular carcinoma, LI-RADS Liver Imaging Reporting and Data System,

CEUS contrast-enhanced ultrasonography, CT computer tomography, MRI magnetic resonance imaging, APHE arterial phase hyperenhancement, AUC area under the curve, PPV positive predictive value, NPV negative predictive value, TP true positive, TN true negative, FP false positive, FN false negative

Table S4 The strengths and drawbacks of CEUS and CT/ MRI in diagnosing recurrent HCC

	CEUS	CT/MRI
Strengths	1. Real-time imaging	1. Superior anatomical detail
	2. High spatial resolution	2. Wide tissue penetration
	3. No radiation exposure and nephrotoxic	3. Multi-planar imaging
	4. Quick image acquisition	
Drawbacks	1. Limited penetration	1. Acquired at a static fixed time (may miss arterial hyperenhancement)
	2. Operator dependence	2. Radiation exposure (for CT)
	3. Short duration of enhancement	3. Time-consuming (for MRI)

HCC hepatocellular carcinoma, CEUS contrast-enhanced ultrasonography, CT computed tomography, MRI magnetic resonance imaging

Table S5 The inter-reader agreement for Liver Imaging Reporting and Data System categories on CEUS, CT and MRI

LI-RADS Categories	Kappa	95% CI
CEUS (*n* = 421)	0.893	0.862, 0.924
CT (*n* = 377)	0.873	0.839, 0.907
MRI (*n* = 91)	0.784	0.663, 0.905

LI-RADS Liver Imaging Reporting and Data System, CEUS contrast-enhanced ultrasonography, CT computed tomography, MRI magnetic resonance imaging, CI confidence intervals
